# Direct-visualization endoscopic retrograde appendicitis therapy for chronic appendicitis with diarrhea: clinical features and outcomes

**DOI:** 10.3389/fmedt.2026.1754047

**Published:** 2026-05-01

**Authors:** Chun Gao, Cheng-Zhou Du, Run-Long He, Wei Wang

**Affiliations:** 1 Department of Gastroenterology, The 940 Hospital of Joint Logistic Support Force of PLA, Lanzhou, Gansu, China; 2 Department of General Surgery, The 940 Hospital of Joint Logistic Support Force of PLA, Lanzhou, Gansu, China

**Keywords:** D-ERAT, chronic appendicitis, treatment, outcomes, appendicitis–diagnosis

## Abstract

**Background and aims:**

Chronic appendicitis complicated with diarrhea presents diagnostic and therapeutic challenges due to its atypical and prolonged symptoms, often mimicking functional gastrointestinal disorders. This study aimed to evaluate the clinical characteristics and therapeutic efficacy of Direct-visualization Endoscopic Retrograde Appendicitis Therapy (D-ERAT) in these patients.

**Methods:**

We retrospectively analyzed 55 patients with chronic appendicitis and diarrhea who underwent D-ERAT under direct endoscopic visualization. Baseline demographic, clinical, and laboratory data, imaging findings, intraoperative endoscopic observations, and postoperative recovery indicators were collected. Diarrhea frequency, stool consistency, inflammatory markers, postoperative complications, and quality of life were assessed. Patients were followed up at 1, 3, and 6 months postoperatively.

**Results:**

Preoperatively, patients experienced a mean of 7.5 ± 2.5 diarrhea episodes per day, with Bristol stool types 5–7. Abdominal CT identified appendiceal abnormalities in all patients, while ultrasound detected 69.1%. Endoscopic findings revealed intraluminal fecaliths in 91.5% and mucosal fibrosis in 87.27%. The patency of the appendiceal lumen was well restored in all cases after the operation. Postoperatively, diarrhea frequency decreased to 1.5 ± 0.4/day, and stool consistency normalized to Bristol types 3–4. Inflammatory markers, including WBC, neutrophil percentage, and CRP, significantly improved. At 6 months, 85.5% of patients remained largely asymptomatic, indicating stable medium-term efficacy. No major adverse events occurred.

**Conclusions:**

D-ERAT is a safe and effective minimally invasive therapy for chronic appendicitis with diarrhea, offering rapid symptom relief, preserved appendiceal function, and shorter hospitalization.

## Introduction

Chronic appendicitis is a relatively common inflammatory condition characterized by fibrosis and hyperplasia of the appendiceal wall, lymphocytic infiltration, and luminal stenosis or occlusion. While the diagnosis and treatment of acute appendicitis are well established, the clinical manifestations of chronic appendicitis are often nonspecific and may mimic conditions such as irritable bowel syndrome, Crohn's disease, or chronic colitis, leading to misdiagnosis or delayed treatment (1, 2). Some patients present with diarrhea, which may be related to gut microbiota dysbiosis, local neural reflexes, or secondary intestinal dysfunction, although the underlying mechanisms remain unclear (3). Diagnosis is currently based on clinical features, imaging (ultrasonography or computed tomography), and histopathology, but there is no universally accepted gold standard (4). In addition to recurrent right lower quadrant pain, atypical symptoms such as diarrhea and abdominal distension are frequently misattributed to functional gastrointestinal disorders. The pathogenesis of diarrhea in chronic appendicitis may involve altered intestinal motility through neuroimmune regulation, disruption of mucosal permeability by inflammatory mediators, and gut microbiota imbalance due to appendiceal dysfunction (5, 6). However, research on chronic appendicitis complicated by diarrhea remains scarce, and clinical management largely relies on empirical anti-inflammatory therapy or surgery.

Inspired by endoscopic retrograde cholangiopancreatography for acute obstructive suppurative cholangitis, Liu Bingrong performed the first case of endoscopic retrograde appendicitis therapy (ERAT) in 2009, which was presented at Digestive Disease Week in 2011 and first published in *Gastrointestinal Endoscopy* in 2012 (7). ERAT is a minimally invasive, organ-preserving technique that involves colonoscopic intubation of the appendix, removal of appendicoliths, and saline irrigation of the appendiceal lumen (8). It provides immediate relief of obstruction, preserves appendiceal function, and offers advantages such as minimal trauma, early feeding, rapid recovery, and a low risk of complications. Over the past decade, ERAT has been increasingly adopted in China with favorable short-term outcomes, low recurrence rates, and good safety profiles (9–12). It is particularly suitable for elderly patients, those with comorbidities, or individuals unwilling to undergo surgical resection.

Although laparoscopic appendectomy (LA) remains the standard treatment for chronic appendicitis, it carries potential risks such as anesthesia-related complications, postoperative adhesions, and infections. In contrast, D-ERAT provides a minimally invasive, organ-preserving option for carefully selected patients without perforation or abscess formation. However, large-scale clinical studies are lacking, especially regarding its effectiveness in alleviating diarrhea symptoms. Moreover, appendiceal fibrosis and other pathological changes may influence procedural success, underscoring the need for precise preoperative evaluation.

In summary, the clinical features and optimal treatment strategies for chronic appendicitis with diarrhea remain unclear. This study aimed to evaluate the clinical characteristics, therapeutic outcomes, and prognostic value of D-ERAT in this patient population, with the goal of providing evidence to optimize diagnostic and therapeutic strategies and improve patient quality of life. Future multicenter randomized controlled trials are warranted to confirm the long-term efficacy and refine the indications for D-ERAT.

## Patients and methods

### Study design and ethics

This retrospective study included patients with chronic appendicitis complicated with diarrhea who underwent Direct-visualization Endoscopic Retrograde Appendicitis Therapy (D-ERAT) at the 940th Hospital of the Joint Logistics Support Force between January 2024 and January 2025. The study protocol was approved by the Institutional Review Board (ID:2024KYLL167).

### Diagnostic criteria

According to 《SAGES guideline for the diagnosis and treatment of appendicitis》 (4), a comprehensive diagnosis was made based on the following elements: (1) Clinical symptom: chronic diarrhea (Bristol type 5–7, >3 times/day) lasting >3 months, accompanied by fever, nausea and vomiting; (2) Physical Examination: No spontaneous pain was noted, but mild to moderate deep tenderness was present at the fixed point of the right lower abdomen (McBurney's point); (3) imaging-confirmed appendiceal abnormalities (e.g., wall thickness >3 mm, fecaliths, luminal dilatation, or peripheral exudate); (4) no evidence of other causes of diarrhea, including infections, inflammatory bowel disease, intestinal tuberculosis, or tumors.

### Inclusion criteria

Patients were eligible if they met all of the following: (1) age 18–75 years; (2) Comply with the diagnostic criteria for all of the above; (3) Patients gave informed consent, voluntarily underwent treatment (such as D-ERAT, surgery, etc.) and participated in clinical follow-up; (4) Abdominal CT evaluation indicated that the appendix was not completely occluded, and there was the possibility of intervention treatment.

### Exclusion criteria

Patients were excluded if they had: (1) indications for emergency surgery, including suspected appendiceal perforation, periappendiceal abscess, or diffuse peritonitis; (2) diarrhea with other definite causes, including IBS-D or systemic diseases such as hyperthyroidism, diabetes, or acute infectious enteritis; (3) severe systemic diseases, including advanced cardiac, pulmonary, hepatic, or renal dysfunction, or coagulation disorders; (4) pregnancy or lactation; (5) recent (within 4 weeks) continuous use of antibiotics, probiotics, or potent antidiarrheal drugs; or (6) inability to cooperate with diagnosis, treatment, or follow-up due to cognitive or mental disorders.

### Preoperative preparation

All patients fasted for 6–8 h prior to the procedure and underwent bowel preparation with compound polyethylene glycol electrolyte powder. Procedures were performed under intravenous or local anesthesia.

### D-ERAT procedure

All procedures were conducted by endoscopists with >5 years of colonoscopy experience and ≥100 D-ERAT procedures annually. The procedure included appendiceal intubation, removal of fecaliths, and saline flushing of the appendiceal cavity. The choice of specific technique was based on intraoperative findings. The surgical process specifically refers to a minimally invasive method of relieving appendiceal obstruction and eliminating inflammation through intubation, imaging, stone removal/drainage, and flushing under endoscopic visualization. The instruments used during the surgery is eyeMAX, Micro-Tech Co., Ltd., Nanjing, China.

### Observation indicators

1. **Baseline Data:** demographic information (age, sex, BMI), medical history (duration of diarrhea, prior treatments), laboratory tests (WBC, neutrophil percentage, CRP), and imaging findings (abdominal CT, appendiceal ultrasound).
2. **Intraoperative Data:** appendiceal orifice and lumen appearance, presence of fecaliths, mucosal fibrosis, purulent discharge, anatomical variations, operation method, and operative time (Average operative time: timing from the beginning of intubation into the appendix to the completion of treatment and the end of withdrawal from the appendiceal cavity.)
3. **Postoperative Data:** improvement in diarrhea frequency and stool consistency (Bristol scale), laboratory markers, pain resolution time, length of hospital stay, and adverse events (e.g., bleeding, perforation, infection).
4. **Quality of Life:** functional diarrhea–related symptoms.

### Follow-up

Of all enrolled patients, systematic postoperative care instructions and regular follow-up assessments were implemented. Key components of patient education included guidance on a stepwise dietary transition, temporary activity restrictions, and clear instructions for symptom monitoring. Subsequently, structured follow-ups were conducted at 1, 3, and 6 months post-procedure via outpatient clinic visits or telephone interviews to meticulously document symptom resolution, signs of appendicitis recurrence, and the occurrence of any long-term complications.

### Statistical analysis

Statistical analyses were performed using SPSS 26.0. Normally distributed continuous variables were expressed as mean ± SD and compared using one-way ANOVA. Non-normally distributed variables were presented as median (interquartile range). Categorical variables were expressed as number (percentage) and compared using chi-square or Fisher's exact test. A *P*-value <0.05 was considered statistically significant.

## Results

### Baseline characteristics

A total of 55 patients with chronic appendicitis complicated by diarrhea underwent D-ERAT. The majority were <65 years old (92.7%), and 69.1% were male. Most patients had a BMI within the normal (56.4%) or overweight range (40.0%). The duration of diarrhea was <2 years in 52.7% of patients, 2–5 years in 35.5%, and >5 years in 11.7%. Laboratory tests showed that 81.8% had elevated WBC (≥10 × 10⁹/L) and 83.6% had elevated neutrophil percentages (≥75%). On imaging, appendiceal fecaliths were detected in all patients (100%), luminal dilatation or fluid collection in 56.4%, and blurred periappendiceal fat planes in 49.1% (Table 1).

**Table 1:** Patient baseline characteristics.

Patient details	Total (*n* = 55)
Age, y, *n* (%)	
<65	51 (92.7%)
≥65	4 (7.3%)
Sex, *n* (%)	
Female	17 (30.9%)
Male	38 (69.1%)
Body mass index, kg/m^2^, *n* (%)	
<18.4	2 (3.6%)
18.5−23.9	31 (56.36%)
>24	22 (40.04%)
Duration of diarrhea, y, *n* (%)	
<2	29 (52.72%)
2–5	19 (35.54%)
>5	7 (11.74%)
WBC (×10^9/L), *n* (%)	
≥10	45 (81.81%)
<10	10 (18.19%)
Neu%, *n* (%)	
≥75%	46 (83.63%)
<75%	9 (16.37%)
Imaging findings (CT)	
Appendiceal luminal dilatation or fluid collection	31 (56.4%)
Blurred periappendiceal fat plane	27 (49.1%)
Appendiceal fecalith	55 (100%)
Appendiceal fecaliths or luminal dilatation (Ultrasound)	38 (69.10%)

### Intraoperative findings

**Figure 1:**
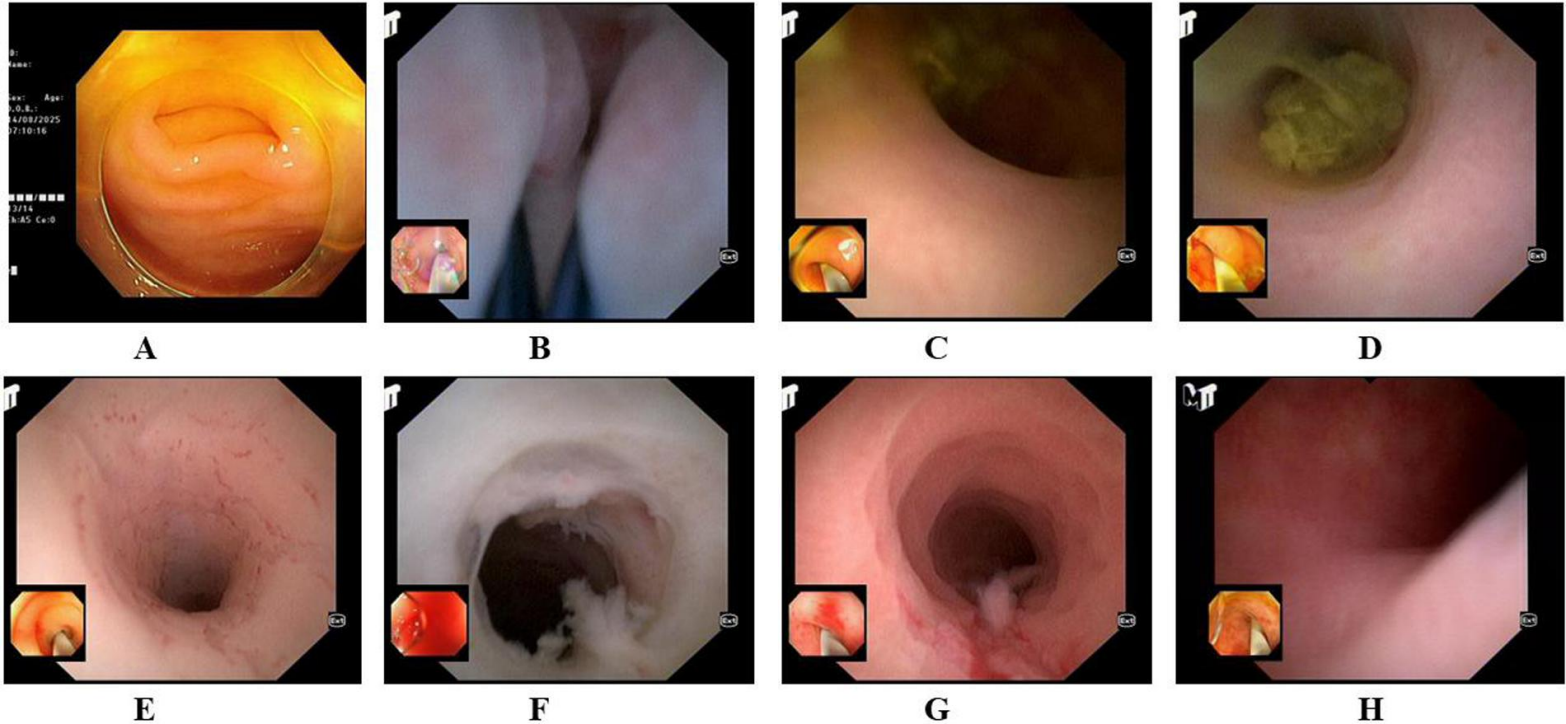
Endoscopic appearance [**(A)** normal opening of the appendix; **(B)** narrowed opening of the appendix; **(C)** fecalith at the opening of the appendix; **(D)** fecalith in the lumen of the appendix; **(E)** narrowed lumen of the appendix; **(F)** pus in the lumen of the appendix; **(G,H)** mucosal fibrosis in the lumen of the appendix].

Endoscopic evaluation of the appendiceal orifice revealed stenosis or occlusion in 16.4% and purulent discharge in 9.1%, while 74.5% appeared normal. Within the lumen, intraluminal fecaliths were observed in 91.5%, mucosal fibrosis in 87.3%, and luminal stenosis in 20.0% (single or multiple). Three patients (5.5%) had a normal intraluminal appearance. D-ERAT combined with eyeMAX was performed in all patients with a median operative time of 7 min (IQR 7.3–10.4) (Table 2, Figure 1).

**Table 2:** Intraoperative endoscopic findings.

Findings	Total (*n* = 55)
Endoscopic appearance of the appendiceal orifice, *n* (%)	
Stenosis or occlusion	9 (16.4)
Impacted fecalith	0
Normal	41 (74.5)
Erosion or ulceration	0
Anatomical variations (high, low, or left-sided appendix)	0
Purulent discharge	5 (9.1)
Endoscopic appearance of the appendiceal lumen, *n* (%)	
Lumen stenosis	
Multiple luminal stenoses	2 (3.6)
Single luminal stenosis	7 (16.4)
Intraluminal fecaliths	52 (91.5)
Normal lumen	3 (5.5)
Intraluminal foreign body	1 (1.8)
Lumen with white pus	7 (12.7)
Mucosal fibrosis	48 (87.27)
Operation method, *n* (%)	
D-ERAT (eyeMAX system)	55 (100.0)
Operation time, min, median (IQR)	7.0 (7.30, 10.41)

### Postoperative recovery

Diarrhea frequency significantly decreased from a preoperative mean of 7.5 ± 2.5 episodes/day to 1–2 episodes/day postoperatively. Stool consistency improved from Bristol stool types 5–7 to types 3–4. Laboratory parameters also showed improvement, with reductions in WBC [from 6.25 (5.82–7.15) to 4.35 (3.89–5.63)], neutrophil percentage [from 64.2% (59.3–71.0%) to 40.3% (35.1–52.6%)], and CRP (from 1.32 ± 1.38 to 0.43 ± 0.62) (Table 3).

**Table 3:** Postoperative outcomes.

Variable	preoperative	postoperative
Diarrhea frequency, times/24 h	5–10	1–2
Stool consistency, Bristol type	5–7	3–4
Laboratory markers		
WBC (×10^9/L), median (IQR)	6.25 (5.82, 7.15)	4.35 (3.89, 5.63)
Neu%, median (IQR)	64.15% (59.30%, 70.97%)	40.26% (35.10%, 52.55%)
CRP, (mean ± SD)	1.32 ± 1.38	0.43 ± 0.62

### Follow-up outcomes

At 1 month, 81.8% of patients had diarrhea frequency <3/day, and 87.3% achieved Bristol type 3–4 stools. By 3 months, diarrhea frequency was further reduced (1.7 ± 0.5/day), and 94.5% of patients had Bristol type 3–4 stools. At 6 months, 85.5% remained largely asymptomatic, indicating stable medium-term efficacy (Table 4).

**Table 4:** Postoperative follow-up outcomes.

Time point	Diarrhea resolution, *n* (%)	Defecate frequency (times/day)	Bristol type 3–4, *n* (%)	Overall response rate (ORR), *n* (%)
preoperative	0	7.5 ± 2.5	0	0
1 month postoperatively	45 (81.8)	2.1 ± 0.8	48 (87.3)	50 (90.9)
3 month postoperatively	50 (90.9)	1.7 ± 0.5	52 (94.5)	53 (96.4)
6 month postoperatively	47 (85.5)	1.5 ± 0.4	51 (92.7)	52 (94.5)

## Discussion

The pathogenesis of acute appendicitis is well recognized; however, the concept of chronic appendicitis remains controversial and unfamiliar to many clinicians. Clear diagnostic criteria are lacking, and its pathophysiology is not fully understood. Earlier reports suggested that recurrent abdominal pain lasting more than 7 days may differentiate chronic from acute appendicitis (13). In reality, chronic appendicitis often presents with persistent but mild abdominal discomfort lasting for weeks to years, far exceeding the natural course of acute appendicitis (14). Because of its insidious and nonspecific symptoms, chronic appendicitis is frequently misdiagnosed or diagnosed late. This diagnostic challenge is particularly evident in patients presenting with diarrhea, in whom the symptoms are often attributed to functional bowel disorders such as irritable bowel syndrome (IBS). Such overlap further obscures the underlying cause of appendiceal inflammation and complicates both diagnosis and treatment. By characterizing the clinical features of patients with chronic appendicitis and diarrhea, our study provides additional insights that may improve diagnostic recognition of this entity.

The underlying mechanism of chronic appendicitis with diarrhea may be explained by disruption of the appendix–microbiota–immune axis. Far from being a vestigial organ, the appendix is now recognized as an important reservoir for gut microbiota and a site rich in lymphoid tissue that maintains mucosal immune balance (3, 15). Chronic inflammation, often triggered by luminal obstruction from fecaliths, lymphoid hyperplasia, or fibrosis, leads to increased intraluminal pressure, bacterial overgrowth, and persistent low-grade inflammation (16). This inflammatory state disrupts immune homeostasis, driving continuous release of proinflammatory cytokines such as IL-17, IL-23, and TNF-α (17, 18). These mediators not only sustain local appendiceal inflammation but also alter the distal intestinal immune environment through systemic and lymphatic pathways. Consequently, gut microbiota dysbiosis occurs, characterized by a loss of beneficial commensals (e.g., short-chain fatty acid–producing Firmicutes) and expansion of potentially pathogenic Proteobacteria (19, 20). In parallel, the colonic mucosa develops low-grade inflammation, increased permeability, and visceral hypersensitivity—pathological features that strongly overlap with diarrhea-predominant IBS (IBS-D) (21). This overlap explains why chronic appendicitis with diarrhea is frequently mistaken for functional bowel disease (37). Importantly, by relieving obstruction and evacuating intraluminal contents, Direct-visualization Endoscopic Retrograde Appendicitis Therapy (D-ERAT) may not only resolve local lesions but also attenuate systemic immune activation, restore microbial balance, and effectively relieve diarrhea.

In the preliminary phase of this study, 30.9% of patients with chronic appendicitis and diarrhea were initially misdiagnosed with functional gastrointestinal disorders such as IBS-D, underscoring the diagnostic challenge. Two major factors contributed to this misdiagnosis. First, clinical manifestations were atypical. Most patients did not present with classic migratory right lower quadrant pain but instead reported nonspecific symptoms such as persistent abdominal distension, irregular abdominal pain, and diarrhea, which closely resemble functional bowel disease. Second, imaging accuracy varied. In our cohort, the sensitivity of ultrasonography was only 69.1%, markedly lower than the 100% sensitivity of abdominal CT. Limitations of ultrasonography included poor visualization of the appendix, high operator dependence, and interference from intestinal gas. To improve diagnostic accuracy, we propose a three-step strategy: (1) structured questionnaires with focused inquiry on symptom exacerbation after high-fat meals; (2) prioritization of abdominal CT to leverage its high spatial resolution and objectivity in assessing appendiceal morphology; and (3) diagnostic antimicrobial therapy (e.g., a one-week course of ciprofloxacin combined with metronidazole), with clinical improvement supporting the diagnosis of appendiceal diarrhea. This systematic approach may reduce misdiagnosis and treatment delays (22, 23).

Endoscopic examination revealed characteristic changes of the appendiceal orifice and lumen that are critical for both diagnosis and therapeutic planning (21). In our cohort, the appendiceal orifice most often appeared normal (74.5%), followed by narrowing (16.4%) or purulent discharge (9.1%). Intraluminal findings included fecaliths (91.5%) and fibrotic, scar-like mucosal changes (87.3%). Imaging supported these findings, with CT demonstrating appendiceal wall thickening (>2 mm) in all cases, luminal dilatation or effusion in 56.4%, and blurred pericecal fat planes in 49.1%. These morphological changes reflect repeated cycles of inflammation and obstruction. Fecalith impaction, lymphoid hyperplasia, and prior infection drive fibrotic remodeling, fibroblast activation, and collagen deposition, leading to luminal stenosis and reduced compliance (24, 25). At the molecular level, this process involves an “inflammation–fibrosis axis” in which infiltrating macrophages and lymphocytes secrete cytokines such as TNF-α, IL-6, and TGF-β. TGF-β, in particular, promotes myofibroblast differentiation through Smad signaling, accelerating extracellular matrix deposition and wall thickening (26, 27). Chronic inflammation also induces epithelial remodeling and fibrotic contraction at the appendiceal orifice, impairing drainage and perpetuating the cycle of “obstruction–inflammation–fibrosis” (28). Morphological assessment by endoscopy therefore not only aids diagnosis but also provides essential anatomical information for targeted interventions such as D-ERAT.

ERAT demonstrated significant efficacy in relieving diarrhea symptoms associated with chronic appendicitis, and our findings provide mechanistic insights into this effect. By relieving appendiceal obstruction (stone removal success rate: 100%), the mean diarrhea frequency decreased from 7.5 ± 2.5 episodes/day preoperatively to 1.5 ± 0.5 episodes/day postoperatively. This suggests that decompression of intraluminal hypertension rapidly alleviates reflex-driven intestinal hyperperistalsis (16). In addition, intraoperative lavage and local antibiotic infusion effectively reduced systemic inflammation, with CRP decreasing from 1.32 ± 1.38 mg/L preoperatively to 0.43 ± 0.62 mg/L postoperatively. D-ERAT thereby removed both the infectious nidus and the obstruction, leading to rapid resolution of local and systemic inflammation, consistent with prior reports that anti-inflammatory therapy promotes rapid mucosal recovery (29, 30). Clinically, this was reflected in normalization of stool form, with most patients achieving Bristol type 3–4 within one week after surgery (31). These results suggest that D-ERAT restores both local appendiceal function and overall gut barrier integrity.

All 55 patients in this study completed scheduled follow-up, with sustained improvement in diarrhea symptoms after D-ERAT. At one month, 81.8% (45/55) achieved normalization of bowel frequency (<3 times/day) with stool consistency restored to Bristol type 3–4. At three months, the response rate further increased to 90.9% (50/55). By six months, 85.5% (47/55) of patients remained asymptomatic or largely symptom-free (defined as ≤2 bowel movements/day and Bristol ≤4), demonstrating durable short- to mid-term efficacy. These findings support DERAT as a minimally invasive, effective treatment for chronic appendicitis complicated by diarrhea, capable of providing both rapid symptom relief and stable outcomes over at least six months.

Our study had several limitations. First, although D-ERAT significantly alleviated diarrhea symptoms, the absence of dynamic gut microbiota monitoring (e.g., pre- and postoperative fecal metagenomic sequencing) limited direct evaluation of the “appendiceal microbiota reservoir” recovery mechanism. Efficacy could only be inferred indirectly through clinical indicators such as diarrhea frequency, which may underestimate the contribution of the microbiota–immune axis to long-term outcomes (32). Second, the single-center retrospective design and relatively small sample size reduced statistical power, particularly for subgroup analyses and assessment of recurrence risk factors. Third, the median follow-up of six months may be insufficient to assess long-term outcomes, as prior literature indicates that recurrence peaks 18–24 months postoperatively (33).

Despite the advantages of D-ERAT—including rapid symptom relief and minimal invasiveness—precise obstruction relief remains critical for durable efficacy (34, 35). Technical limitations also exist: intubation failure occurs in approximately 5%–15% of cases, mainly due to anatomical variations of the appendiceal orifice, and transient bacteremia is observed in 3%–8% of patients, typically resolving spontaneously or with short-term antibiotic therapy. Risk mitigation requires strict indication screening (e.g., preoperative CT confirmation of appendiceal obstruction or fecaliths), adherence to perioperative aseptic protocols, judicious use of prophylactic antibiotics, and close postoperative monitoring of vital signs (36).

Future studies should focus on multicenter, prospective designs incorporating metagenomic and immunohistochemical analyses to systematically evaluate D-ERAT-mediated remodeling of the appendiceal microbiota and intestinal mucosal barrier. Such investigations could elucidate long-term efficacy, recurrence predictors, and the mechanistic interplay between local appendiceal pathology and systemic gastrointestinal function.

## Conclusion

Patients with chronic appendicitis complicated by diarrhea often face diagnostic challenges, as their symptoms overlap with functional gastrointestinal disorders such as irritable bowel syndrome. The underlying mechanism likely involves disruption of the neuro-immune-microbiota axis triggered by chronic appendiceal inflammation. Direct-visualization Endoscopic Retrograde Appendicitis Therapy (D-ERAT) effectively relieves appendiceal obstruction, controls local inflammation, and preserves organ integrity, resulting in rapid alleviation of diarrhea and other clinical symptoms. Compared with conventional laparoscopic surgery, D-ERAT offers advantages including shorter hospitalization and reduced overall medical costs. Limitations of this study include the absence of dynamic gut microbiota monitoring, which precluded direct confirmation of D-ERAT-induced microbiota remodeling. Future studies employing multi-omics approaches are warranted to elucidate the mechanisms of host-microbiota interactions. Based on current evidence, D-ERAT can be considered a first-line minimally invasive treatment for carefully selected patients, particularly elderly individuals or those with comorbidities, provided strict adherence to indications and standardized operative procedures to minimize recurrence and complications.

## Data Availability

The original contributions presented in the study are included in the article/Supplementary Material, further inquiries can be directed to the corresponding authors.
